# Comparisons of microRNA Patterns in Plasma before and after Tumor Removal Reveal New Biomarkers of Lung Squamous Cell Carcinoma

**DOI:** 10.1371/journal.pone.0078649

**Published:** 2013-10-09

**Authors:** Vasily N. Aushev, Irina B. Zborovskaya, Konstantin K. Laktionov, Nicolas Girard, Marie-Pierre Cros, Zdenko Herceg, Vladimir Krutovskikh

**Affiliations:** 1 Epigenetics Unit, International Agency for Research on Cancer (IARC), Lyon, France; 2 Carcinogenesis Institute of N.N Blokhin Russian Cancer Research Center, Russian Academy of Medical Sciences, Moscow, Russia; 3 Department of Respiratory Medicine, National Expert Centre for Thymic Malignancies, Reference Centre for Orphan Pulmonary Diseases, Louis Pradel Hospital, Hospices Civils de Lyon, Lyon, France; University of Pittsburgh, United States of America

## Abstract

Lung cancer is the major human malignancy, accounting for 30% of all cancer-related deaths worldwide. Poor survival of lung cancer patients, together with late diagnosis and resistance to classic chemotherapy, highlights the need for identification of new biomarkers for early detection. Among different cancer biomarkers, small non-coding RNAs called microRNAs (miRNAs) are considered the most promising, owing to their remarkable stability, their cancer-type specificity, and their presence in body fluids. However, results of multiple previous attempts to identify circulating miRNAs specific for lung cancer are inconsistent, likely due to two main reasons: prominent variability in blood miRNA content among individuals and difficulties in distinguishing tumor-relevant miRNAs in the blood from their non-tumor counterparts. To overcome these impediments, we compared circulating miRNA profiles in patients with lung squamous cell carcinoma (SCC) before and after tumor removal, assuming that the levels of all tumor-relevant miRNAs would drop after the surgery. Our results revealed a specific panel of the miRNAs (miR-205, -19a, -19b, -30b, and -20a) whose levels decreased strikingly in the blood of patients after lung SCC surgery. Interestingly, miRNA profiling of plasma fractions of lung SCC patients revealed high levels of these miRNA species in tumor-specific exosomes; additionally, some of these miRNAs were also found to be selectively secreted to the medium by cultivated lung cancer cells. These results strengthen the notion that tumor cells secrete miRNA-containing exosomes into circulation, and that miRNA profiling of the exosomal plasma fraction may reveal powerful cancer biomarkers.

## Introduction

Lung cancer is the major cause of cancer mortality worldwide [1]. This is largely due to the lack of efficient tools for early diagnosis of the disease. Although different approaches (spiral computed tomography, promoter hypermethylation analysis, etc.) have been extensively tested in observational trials of lung cancer, most of these methods seem to be inefficient for early diagnosis of asymptomatic lung tumors. In particular, for individuals at high risk of developing lung cancer, such as current and former smokers, the development of effective blood-based assays remains of paramount importance.

Early detection is particularly crucial for tumors without clinical manifestations during the initial stages of their development, among which lung cancer holds a special place. In certain forms, namely lung squamous cell carcinoma (SCC), it is asymptomatic until it reaches a significant size, at which point surgery is often inefficient. In addition, smoking plays a key role in the etiology of this type of tumor. Importantly, not all smokers develop lung cancer. Therefore, to identify smokers with a high risk of lung cancer, highly specific and sensitive tests are required.

The major obstacle in achieving this goal is the absence of reliable and simple diagnostic tests based on cancer biomarkers. An ideal biomarker should have several features, including high stability and specificity, and ease of detection in biofluids. To date, all known cancer biomarkers are either unstable or not specific enough to match these basic criteria. The situation has changed recently with the discovery of small non-coding RNAs called microRNAs (miRNAs), a new class of post-translational regulators of protein synthesis whose deregulation is considered to be an important mechanism underlying cancer development [2,3]. Through their direct control of the expression of nearly all proteins, miRNAs are involved in virtually all physiological and pathological processes, including carcinogenesis. Owing to their distinct pattern of expression in a tumor-specific and tumor subtype-specific manner, miRNAs are considered highly attractive targets for biomarker discovery [4].

Furthermore, it has been shown that significant amounts of miRNAs can be found in blood [5] and that such circulating miRNAs can be tumor-specific [6]. Importantly, circulating tumor-specific miRNAs are extremely stable, a feature that makes them reliable and robust biomarkers to detect cancer through a minimally invasive blood test (“liquid biopsy”) [7]. The stability of circulating miRNAs can be explained by their incorporation into small membrane vesicles of endocytic origin called exosomes [8].

Indeed, numerous studies aimed at identifying cancer-specific circulating miRNA profiles have recently been undertaken for many cancer types [9-12]; however, the results obtained so far are disappointingly inconsistent [2]. This may be due to the capacity of miRNAs to reflect the tiny patho-etiological nuances of individual neoplasms, along with the heterogeneity of sets of tumors taken for analysis, the insufficient “purity” of analyzed tumor tissue, the different study designs used, or the different analytical platforms used in each study. The criteria for interpretation and normalization of the obtained results as well as the availability of adequate normal tissue controls could also have contributed to these inconsistencies.

Cancer-specific miRNAs found in body fluids represent attractive targets for the development of blood-based tests for early diagnosis of lung cancer. The search for lung cancer-specific miRNAs, both in primary tumors and in the blood of cancer patients, was performed in several recent studies [6,8,13-22]. Unfortunately, the results obtained were inconsistent and further studies are required for identification and validation of miRNAs before the eventual introduction of miRNA-based tests in screening programs. One of the reasons for these failures is the fact that a pathologically and etiologically heterogeneous group of lung cancers, commonly known as non-small cell lung cancer (NSCLC), were used in miRNA profiling. Another common weakness of previous studies is the lack of intrinsic controls that could be used as reference points to reveal tumor-caused deregulation of individual miRNAs. Instead, healthy individuals were usually used as controls, and a large number of samples would thus be required to make up for pronounced inter-individual variability in the patterns of circulating miRNAs. Therefore, further studies with a design free of the above-mentioned shortcomings are required to reveal the full diagnostic potential of circulating miRNAs for lung cancer.

In this study, we aimed to identify circulating miRNAs specific to lung SCC, one of the most frequent histological types of lung cancer, with a particular emphasis on the screening value of miRNAs to detect this type of tumor at the preclinical stage among high-risk subjects. To overcome previous limitations in this setting, we compared circulating miRNA profiles in patients with lung SCC before and after tumor removal. In addition, we performed miRNA profiling of the exosomal and exosome-free fractions of lung cancer patients with lung SCC. This approach allowed us to identify lung SCC-specific miRNA markers in both plasma and tumor-specific exosomes.

## Materials and Methods

### Sample collection

Blood samples were collected at the N.N. Blokhin Cancer Research Center, Moscow, Russian Federation, and at the Hôpital Louis Pradel, Hospices Civils de Lyon, France. For sample selection, we focused on patients diagnosed with early (T1 or T2) lung SCC. However, some of the patients were reclassified as T3 after further verification. All participating patients signed informed consent, and the study was approved by the institutional ethics review committee of the International Agency for Research on Cancer (IARC). The clinico-morphological stages of tumors were determined according to the standard TNM classification systems of the International Union Against Cancer (6th edition). Systematic pathological review was performed. Blood samples were collected twice: a day before medical preparation for the surgical intervention and then 7–10 days after the surgery, usually on the day of discharge from the hospital. For each sample, 6–10 mL of blood was taken in EDTA-containing Vacuette tubes and centrifuged to separate the plasma fraction. Plasma was then stored at –80°C until further processing.

### RNA isolation and processing

RNA from plasma samples was isolated by using NucleoSpin miRNA Plasma columns (Macherey-Nagel, Germany), with addition of spike-in miRNA (cel-miR-39). The quality of extracted RNA was analyzed by reverse transcription (RT) using the Universal cDNA Synthesis kit (Exiqon, Denmark), and complementary DNA (cDNA) after dilution was used for further analysis. The quality and quantity of isolated RNA were controlled by an Agilent 2100 Bioanalyzer (Agilent Technologies) as described previously [23].

### miRNA profiling

Although the results of previous studies to identify circulating miRNAs specific to lung cancer were rather inconsistent, they nevertheless detected a large spectrum of individual miRNA species deregulated in the blood and primary lung tumors. Based on these findings, we compiled a list of 90 miRNAs, whose expression has already been reported as deregulated in lung and other cancers (Table 1). miRCURY LNA detection probes (Exiqon) specific for each selected miRNA were used for RT polymerase chain reaction (PCR) miRNA profiling. cDNA of each examined sample was distributed in 96 wells of 384-well panels containing these 90 miRNA probes along with several control probes, including small nucleolar RNAs and internal calibrators (Table 1), and processed on an ABI 7900HT system (Applied Biosystems) following the manufacturer’s instructions. Raw data were analyzed using SDS software (Applied Biosystems), and C_t_ values were used as a read-out.

**Table 1:** List of miRNA probes used to profile plasma samples from lung SCC patients.

hsa-let-7a	hsa-miR-20b	hsa-miR-92a	hsa-miR-155	hsa-miR-221	hsa-miR-486-5p
hsa-let-7b	hsa-miR-21	hsa-miR-93	hsa-miR-191	hsa-miR-222	hsa-miR-493
hsa-let-7c	hsa-miR-24	hsa-miR-1254	hsa-miR-192	hsa-miR-223	hsa-miR-496
hsa-let-7d	hsa-miR-25	hsa-miR-98	hsa-miR-194	hsa-miR-324-3p	hsa-miR-497
hsa-let-7e	hsa-miR-26a	hsa-miR-103	hsa-miR-195	hsa-miR-324-5p	hsa-miR-505
hsa-let-7f	hsa-miR-27a	hsa-miR-106a	hsa-miR-196b	hsa-miR-326	hsa-miR-518b
hsa-let-7g	hsa-miR-27b	hsa-miR-122	hsa-miR-197	hsa-miR-335	hsa-miR-566
hsa-miR-1	hsa-miR-28-3p	hsa-miR-130b	hsa-miR-199a-5p	hsa-miR-346	hsa-miR-605
hsa-miR-10a	hsa-miR-29a	hsa-miR-132	hsa-miR-200c	hsa-miR-365	hsa-miR-638
hsa-miR-10b	hsa-miR-29b	hsa-miR-133b	hsa-miR-202	hsa-miR-375	hsa-miR-660
hsa-miR-15a	hsa-miR-29c	hsa-miR-140-5p	hsa-miR-204	hsa-miR-378	SNORD38B
hsa-miR-15b	hsa-miR-30a	hsa-miR-141	hsa-miR-205	hsa-miR-422a	SNORD49A
hsa-miR-16	hsa-miR-30b	hsa-miR-143	hsa-miR-206	hsa-miR-423-5p	U6 snRNA
hsa-miR-19a	hsa-miR-30c	hsa-miR-145	hsa-miR-210	hsa-miR-432	
hsa-miR-19b	hsa-miR-30d	hsa-miR-150	hsa-miR-212	hsa-miR-451	
hsa-miR-20a	hsa-miR-22	hsa-miR-152	hsa-miR-214	hsa-miR-485-3p	

### Validation of selected miRNAs

Based on the data obtained from the initial, discovery step of the study, we performed a validation step for the most promising miRNAs. Validation of miRNAs was done using individual miRNA assays from Exiqon. For this step, the set of analyzed samples was extended: in addition to samples from 32 patients used for profiling, we tested samples from 18 more patients with the same diagnosis and an additional 6 patients without NSCLC. For validation, quantitative PCR (qPCR) was performed in triplicate, and mean values of C_t_ were used for calculation.

### Comparison of exosomal and exosome-free miRNAs from plasma samples

To augment the intensity of tumor-specific miRNA, pre-operative plasma samples from 10 of the previously selected lung SCC patients were used for exosomes precipitation using a polymer-based ExoQuick reagent (SBI, System Biosciences), designed to isolate exosomes, according to the manufacturer’s protocol. Although ExoQuick does not distinguish tumor exosomes from exosomes produced by normal cells, we assume that the portion of tumor miRNA would be higher in the exosome precipitate than in the whole plasma due to a higher capacity of tumor cells to secrete exosomes. The quantity of total miRNA and profiles of individual miRNAs in the exosomal plasma fraction were compared with those in the residual exosome-free fraction of the same samples using the same panel of 90 miRCURY LNA detection probes (Exiqon) that we used with whole-plasma samples.

### *In vitro* comparison of intracytoplasmic and exosomal miRNA spectra in lung cancer cells

It was found, for certain types of cancer, that the miRNA content of exosomes secreted by the tumor cells differs from the miRNA found in tumor cells themselves [24]. To verify whether this phenomenon occurs in lung cancer, we compared *in vitro* miRNA patterns of lung cancer cells with patterns of exosomes secreted by these cells, by using the A2182 human lung cancer cell line. A2182 cells, taken from the Biobank of IARC, were cultivated as 60-90% monolayer at 37°C with 5% CO_2_ in Dulbecco’s Modiﬁed Eagle Medium (DMEM) with 10% fetal bovine serum (FBS). miRNA was isolated separately from the cell monolayer and from exosomes harvested from culture medium. The miRNA expression profiles in these samples were analyzed using the same 90 miRNA probes (Exiqon) as for plasma samples. Additional experiments included A549 (human lung adenocarcinoma), HEK293 (human embryonic kidney) and HuH7 (human liver carcinoma) cell lines cultured in the same conditions.

## Results

### Study design

To identify circulating cancer-specific miRNAs, we compared miRNA contents in the plasma of patients with operable lung SCC before and after surgical removal of the tumor, assuming that all miRNA species disappearing from the blood after surgery are likely to be tumor-specific. Comparing pairs of pre- and post-operative samples from the same individual excluded the factor of inter-individual variability of expressed miRNAs, but did not exclude the possibility of a post-surgery effect. For our miRNA profiling study, we initially selected 34 patients: 32 patients with lung SCC and 2 patients who underwent the same type of lung surgery due to causes other than lung SCC (one with carcinoid and one with sarcomatoid tumors), who served as additional controls. The lung SCC group included 28 (88%) men and 4 (12%) women, with a median age of 60.9 years (range, 47–79 years). Patient data is summarized in Table S1.

The quality and quantity of isolated RNA were controlled by an Agilent 2100 Bioanalyzer (Figure S1). Because miRNAs could get into the bloodstream not only by physiological release from the cells but also due to undesirable hemolysis of red blood cells during processing of blood samples into the plasma [25,26], the presence of hemolytic miRNAs was examined in all samples by measurement of 415 nm absorption [23]. Samples with hemoglobin content higher than 0.1 g/L were considered as affected by hemolysis and were therefore excluded from further analysis.

#### Overall expression levels of circulating miRNAs

Most miRNAs from the designated list were successfully detected (with C_t_ values between 18 and 35) in virtually all samples. The highest signal was detected for miR-451, -223, -15a, -486-5p, -16, and -21, which is in agreement with literature data [27]. A small portion of analyzed miRNAs (including miR-206, -422a, and -202) have rarely been detected in our samples, and these miRNAs are likely to be present in small quantities or completely absent in plasma. For further analysis, global expression levels for each sample were normalized using the BRB-ArrayTools statistical software package [28] (Figure S2).

### miRNAs from tested panel differentially expressed between pre- and post-operative plasma samples

**Figure 1:**
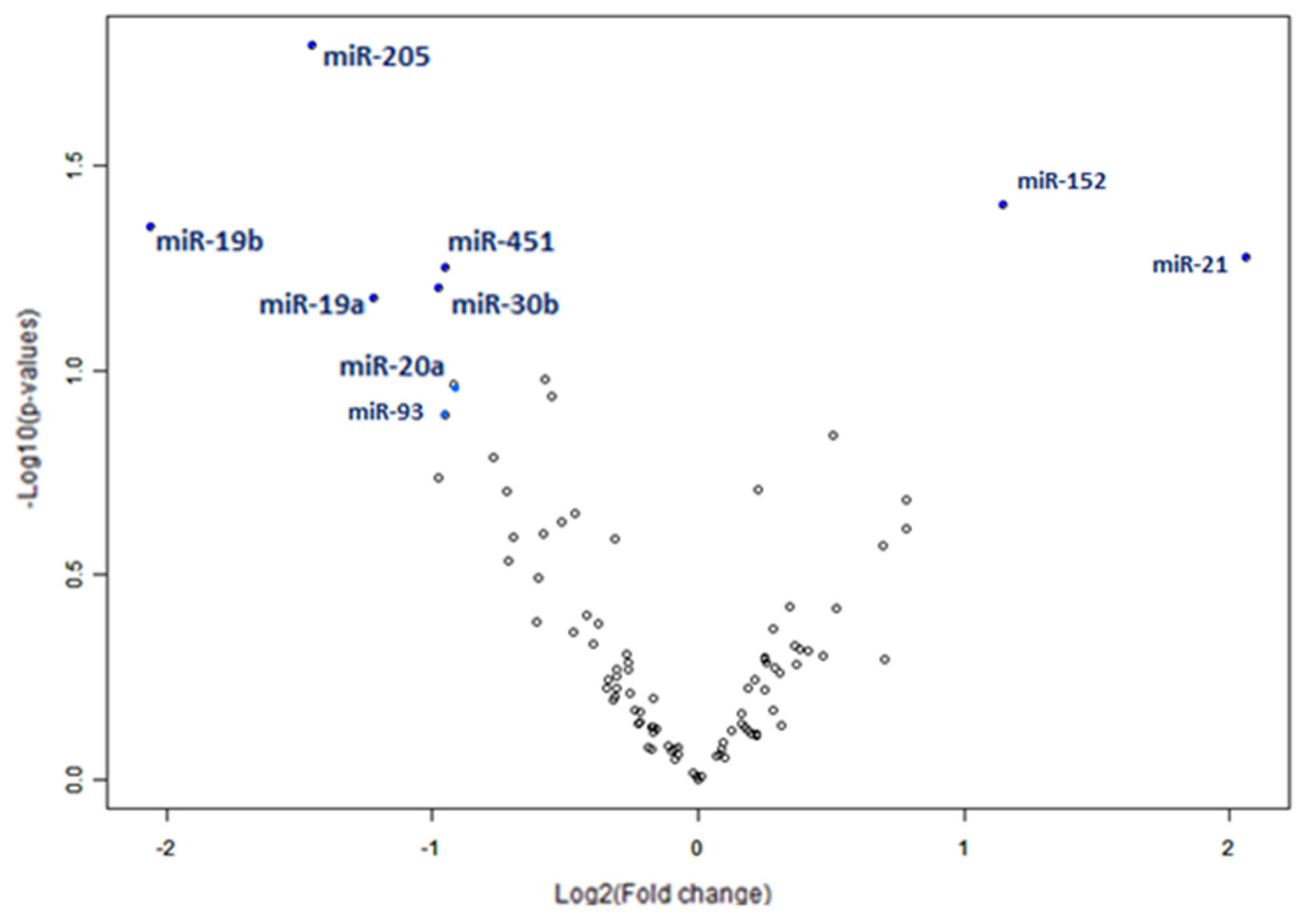
Scatter plot of pre-/post-surgery ratios for expression of the analyzed miRNAs in patients with lung SCC. Analysis was performed with BRB-ArrayTools software. A group of miRNAs on the left side of the plot (miR-205, -19a, -19b, -451, -30b, -20a, and -93) are the most abundant in the plasma of lung SCC patients and are reduced in the plasma after tumor removal.

After initial normalization, we selected miRNAs that displayed the most significant difference between pre- and post-operative samples. Expression of miR-205, -19a, -19b, -20a, -451, and -30b decreased significantly (p<0.05) after tumor removal (Figure 1). Importantly, in two pairs of plasma samples, taken from patients who underwent the same type of lung surgery due to causes other than lung SCC (one with carcinoid and one with sarcomatoid tumors), we did not find any significant post-operative decrease in the levels of these miRNAs. In contrast to some literature data [29], we did not observe significant tumor-associated expression of circulating miR-21.

### Validation of selected candidates

**Figure 2:**
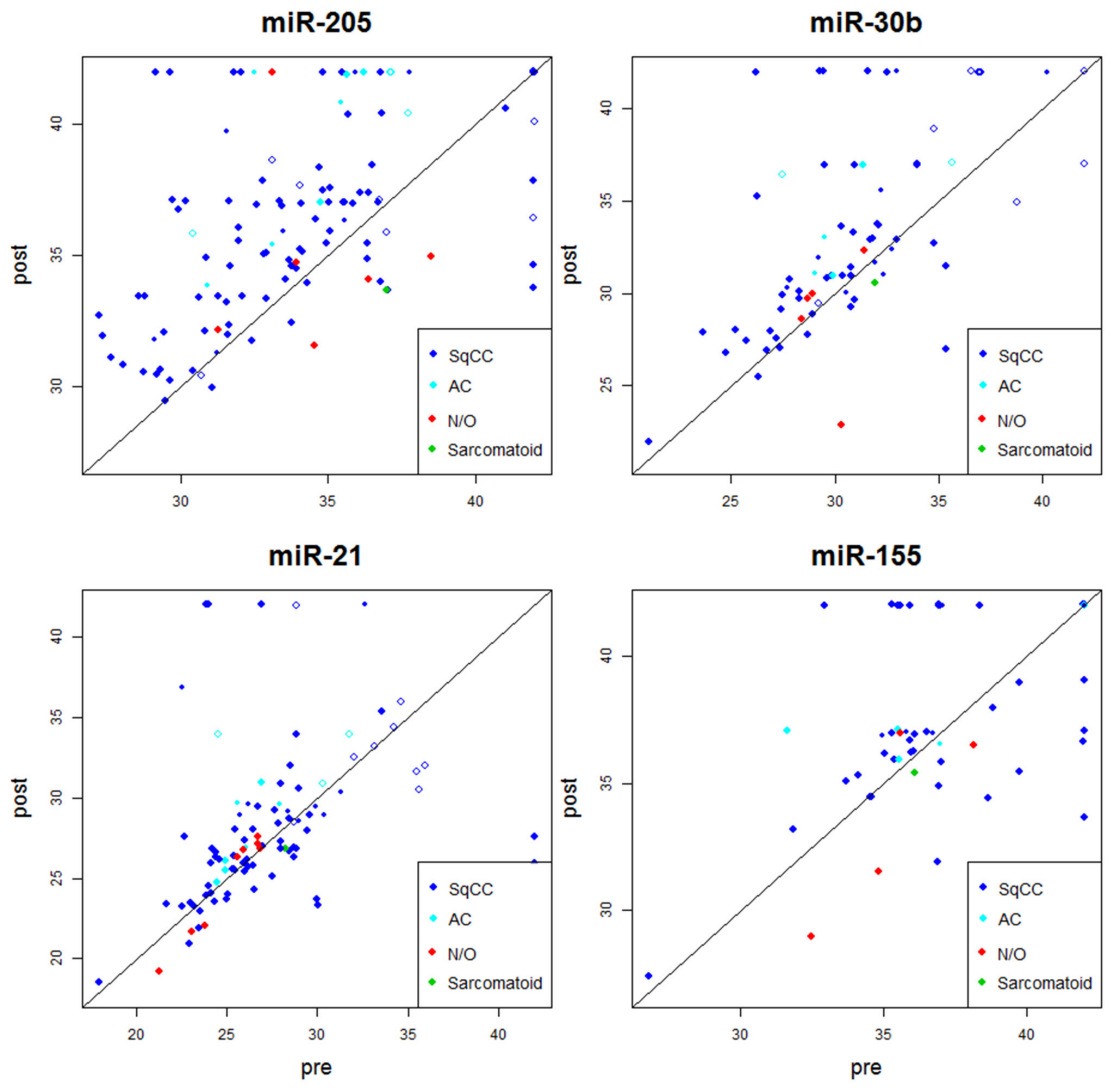
Examples of miRNAs for which levels decreased markedly after tumor removal (upper panel) and of those for which levels did not change significantly (lower panel). SqCC - squamous cell carcinoma, AC – adenocarcinoma, N/O – non-oncological cases.

MiRNAs identified as differentially expressed in the blood after tumor removal were further validated by qPCR, using 18 additional samples. The results of the validation confirmed the indicated changes in miRNA expression. The strongest effect was observed for miR-205: this miRNA was downregulated in the vast majority of cases of lung SCC after tumor removal, but not in the two control cases (Figure 2). Specificity of this result was further confirmed on other non-NSCLC samples: in two esophageal carcinoma cases, one hamartoma case and two healthy donors, we did not observe miR-205 expression in pre-operative samples (Table S2). Other miRNAs downregulated markedly included miR-30b and miR-19, in both miR-19a and miR-19b forms. MiR-21 did not show any significant decrease, confirming our panel-based data. Several miRNAs (e.g., miR-155) were on average upregulated in post-operative samples, but this effect was not specific for oncology patients and thus can be considered to be the result of possible inflammation or another surgery-specific marker.

### miRNA profiles specific to secreted exosomes

MiRNAs in the circulatory system of cancer patients can originate not only from circulating tumor cells but also from exosomes secreted by the primary tumor cancer cells. Furthermore, a significant amount of miRNAs in the plasma is independent of exosomes, but those miRNAs are also stable due to forming a complex with Argonaute-2 (a protein component of the RNA-induced silencing complex [RISC]) or other proteins [30]. Therefore, to better understand the origins of circulating miRNAs, we next characterized the miRNA profiles in circulating exosomes and compared them with those in the exosome-free plasma fraction.

**Figure 3:**
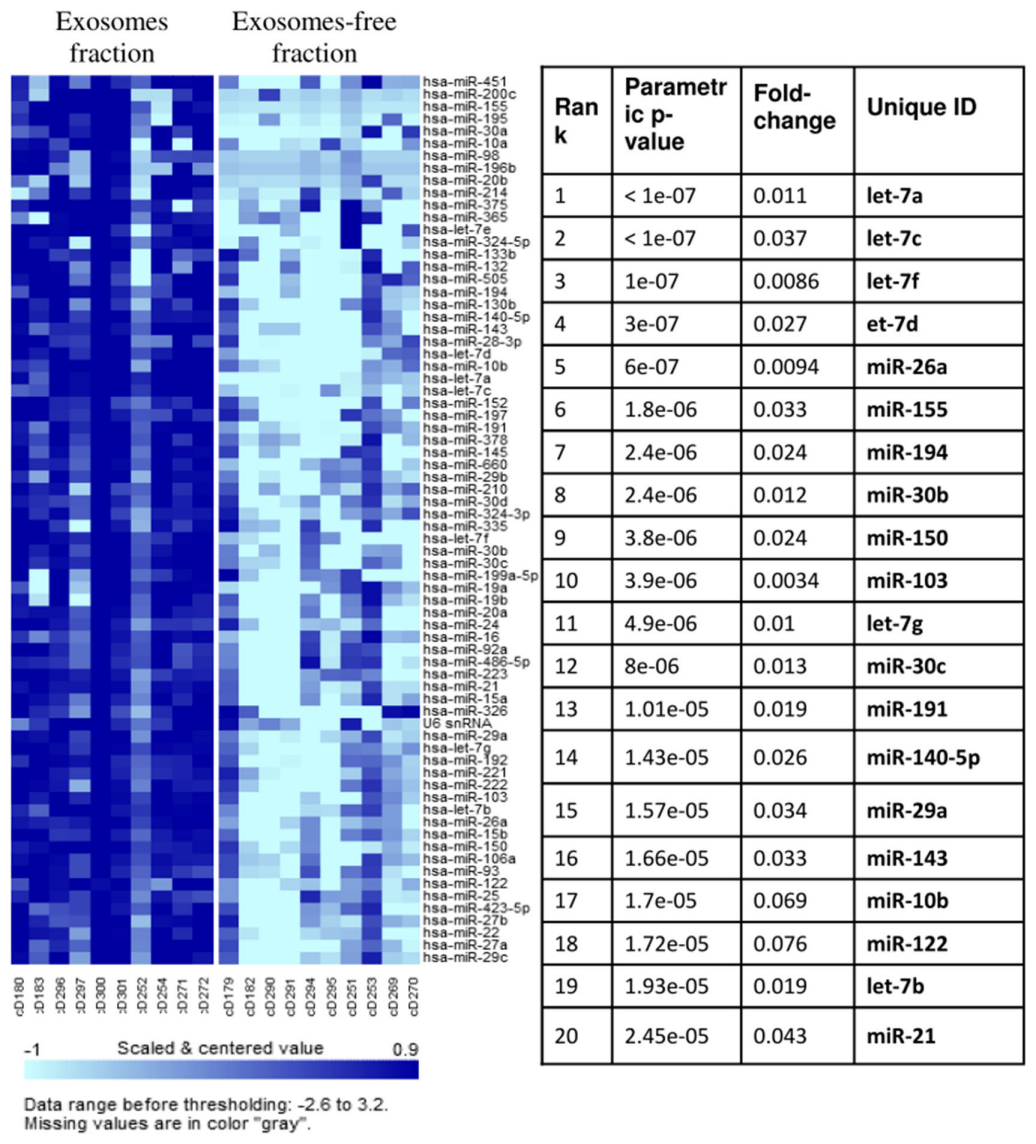
Enrichment in expression of various miRNA species in the ExoQuick-precipitated fraction (“Exosomal fraction”) compared with the ExoQuick-depleted fraction (“Exosome-free fraction”) of the same pre-operative plasma samples from patients with lung SCC.

To this end, the exosomal fraction of the plasma from the lung SCC patients was precipitated by ExoQuick reagent and examined for miRNA levels using the same qPCR approach. We found that the ExoQuick-precipitated fraction was dramatically enriched with most species of miRNAs (Figure 3). Importantly, the exosome-enriched fraction contained most of the miRNAs that we had found to be associated with the presence of the tumor.

### Intracytoplasmic miRNA sorting into exosomes is a selective process in lung cancer cells

**Figure 4:**
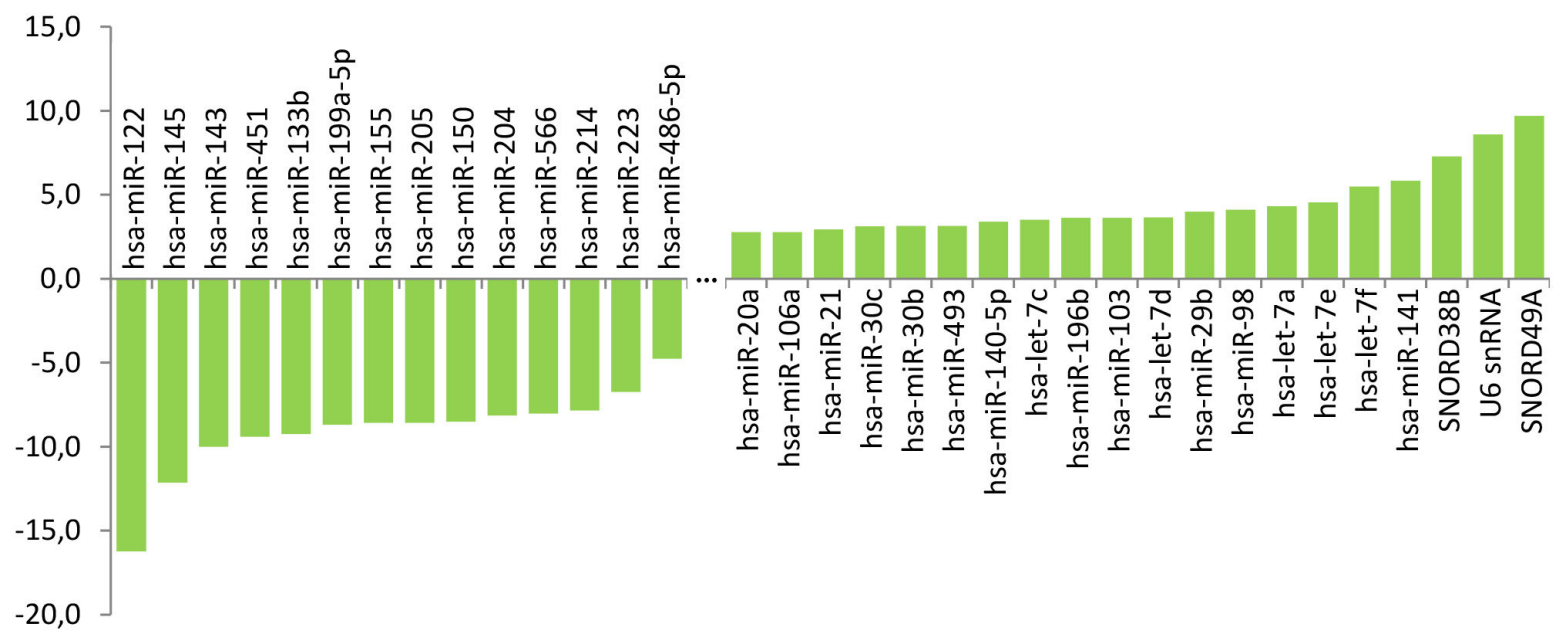
Distribution of miRNA subsets specifically either secreted into the medium (left) or retained in the cytoplasm (right) of the A2182 human lung cancer cell line. Numbers correspond to the logarithm of fold change of miRNA expression level detected in cells versus conditioned medium.

To validate and further expand our findings on exosome-specific miRNAs, we cultured A2182 lung cancer cells *in vitro* and characterized the profile of miRNA secreted from cancer cells. Profiling of the miRNA secreted into the medium by lung cancer cell exosomes and the cytoplasmic miRNA content of these cells revealed subsets of individual miRNAs, which were either selectively enriched in secreted exosomes or preferably retained in the cytoplasm of cancer cells (Figure 4). It is noteworthy that miR-451 and miR-205, two species whose levels were most downregulated in the plasma of lung SCC patients after surgical removal of the tumor, were selectively secreted by lung cancer cells into the medium. Interestingly, these results were cell line-specific: in A549 lung adenocarcinoma cells miR-205 was much less secreted and in HuH7 liver carcinoma cells miR-205 was not detected neither in cells nor in medium; while miR-30b (cell-retained in A2182) was found to be excreted both in A549 and HuH7 (data not shown).

## Discussion

The aim of this study was to identify circulating miRNAs whose levels in blood are associated with the presence of lung SCC. Using a rigorous approach based on comparison of pre- and post-operative miRNA levels, our study has established a lung SCC-specific circulating miRNA signature.

Choosing circulating and not tissue miRNAs as potential biomarkers has some obvious advantages. Although the knowledge of miRNA signatures of certain solid tumors is rather informative in terms of elucidation of the molecular mechanisms involved in their pathogenesis, its diagnostic capacity is limited. To examine a miRNA profile for diagnostic purposes, tumor tissue has to be obtained, either surgically or through invasive procedures such as fine-needle aspiration biopsy (FNAB), which provides a randomly variable yield of tumor cells. Therefore, from a practical point of view, the possibility of detecting tumor miRNAs outside of the primary tumor, in particular in the bloodstream, is the most accessible and simple alternative to diagnose the cancer. Another prominent feature of circulating miRNAs is their remarkable stability [7]. The mechanism behindthis stability are not yet clear: it is proposed, for example, that miRNAs are protected by forming complexes with proteins such as Argonaute [31] and GW182 [32]; encapsulation in exosomes also provides additional protection from degrading enzymes [33]. It should be noted however, that in reality searching blood for tumor-specific miRNAs has proved to be a rather complicated issue. Namely, due to their low levels in circulation it is technically challenging to separate tumor-specific miRNAs from highly abundant miRNAs originating from various non-tumor sources (activated platelets, mononuclear phagocytes, neutrophils, endothelial cells). To overcome this obstacle, specific measures to reveal tumor-relevant miRNAs must be elaborated and implemented.

Another feature of this study was the approach of comparing pre- and post-operative samples. We assumed that most, if not all, tumor-specific miRNA would disappear from the bloodstream soon after surgical eradication of the tumor. This study design retraced the cause-and-effect relationship between the presence or absence of the tumor and circulating tumor-relevant miRNAs, and essentially diminished the undesirable impact of inter-individual variability in circulating miRNA content. The normalization of levels of circulating miRNAs after tumor removal was reported repeatedly for different human neoplasms [34-38].

As a result, we found five miRNA species, miR-205, -19a, -19b, -20a, -451, and -30b, that displayed strong and specific downregulation in plasma 7–10 days after tumor resection. To exclude false positivity due to post-surgical recovery, we compared miRNA profiles in the plasma of lung NSCLC patients after surgery with profiles of two patients who underwent lung surgery for non-lung cancer diseases (carcinoid and sarcomatoid tumors). Importantly, in both of these cases we failed to find any significant changes in the levels of these six miRNAs after the surgery, suggesting that the identified miRNAs are NSCLC-specific.

In our results, miR-205 displayed the most consistent decrease after tumor removal. This miRNA was previously described as specifically overexpressed in SCC [39,40]. In agreement with our results, a recent study reported a similar decrease in miR-205 expression in the serum of lung cancer patients after tumor removal [29]. The latest data confirm that miR-205 expression is increased in tissues and serum for both adenocarcinoma and SCC patients [41]. Another recent study suggests miR-205 as a potential biomarker to differentiate adenocarcinoma from SCC, but only for tissue expression [42]. In our samples, we could not find significant differences in the level of circulating miR-205 between these two subtypes but this can be linked to the low number of adenocarcinoma cases tested. It should also be noted that some lung tumors represent mixed formations containing both squamous cell- and adenomatous structures. As for the biological function of miR-205, this miRNA cannot be unambiguously placed in the cancer development pathway due to its dual role: it can be considered as an oncomiR or a tumor suppressor, depending on the cellular context and signaling pathway. For example, miR-205 was shown to counteract epithelial-mesenchymal transition and tumor invasion, acting as tumor suppressor while numerous other studies show its overexpression in cancer cells [43]. Recent data link miR-205 expression with the tumor suppressor Arf claiming that miR-205 overexpression up-regulates the Arf–p53–p21 signaling axis [44].

MiR-19a and miR-19b belong to the miR-17-92 cluster, also known as oncomiR-1. These miRNAs are believed to play a role in cancer development but initially have not been shown to be lung cancer biomarkers. Recent data, however, link serum miR-19a with bad prognosis in NSCLC [45]. Similarly, miR-30b is also suggested as a possible oncomiR as it was shown to be overexpressed in oral SCC [46] and in the serum of rats bearing pancreatic ductal adenocarcinoma [47].

The value of miR-451 as an oncomarker is questionable. We observed markedly decreased levels of miR-451 after tumor removal, even after exclusion of highly hemolyzed samples. However, miR-451 is known to be expressed at very high levels in red blood cells [26], i.e. significant amounts of miR-451 can be released in cases of slightly hemolyzed samples.

Although precipitation of exosomes from whole plasma by ExoQuick polymer, which we used in this study, does not segregate tumor exosomes from their normal counterparts, it excludes all exosome-free circulating miRNAs, which results in a relative increase in tumor-relevant exosomes. As we showed, the ExoQuick-precipitated exosomal fraction from plasma has a rather high global miRNA concentration and includes most miRNAs identified in whole plasma as lung SCC-specific. This technique does not exclude the presence of other tumor miRNAs in the circulation, independent of exosomes. Additional studies are needed to scrutinize this possibility.

One of the most intriguing observations repeatedly reported for different types of human cancer is the prominent difference between miRNA profiles in primary tumors and the tumor-specific plasma miRNA content of the same cancer patients [48]. This could be explained, at least in part, by selective sorting of cytoplasmic miRNA content of tumor cells into exosomes. This possibility was first shown experimentally for normal and malignant mammary cell lines [49]. Here, we demonstrated for cultured cells that lung cancer cells exhibit the same feature. It is noteworthy that miR-451 and miR-205, which we found among the most downregulated miRNAs in the plasma of lung SCC patients after surgical removal of the tumor, are selectively secreted by lung cancer cells into the medium. This finding also explains why let-7 family miRNAs, described repeatedly as specific for lung cancer tissue (and detected in our study among the miRNAs selectively retained in the cytoplasm of lung cancer cells) were not found at increased levels in the blood of lung cancer patients and thus could not be used as lung cancer biomarkers in a blood test system. To better understand the nature and biological meaning of this phenomenon, miRNA profiles of primary tumors should be compared with those of circulating tumor exosomes in different types of cancer and at different stages of development.

In conclusion, our study identified lung SCC-specific miRNAs in plasma by profiling a large set of miRNAs in the blood of patients before and after tumor removal. Our results suggest a potential diagnostic value of a panel of miRNAs (miR-205, -19a, -19b, -30b, -20a) as blood-based markers. Further studies are needed to validate the clinical utility of these circulating miRNAs. A larger cohort with homogeneous sets of tumors combined with next-generation sequencing would offer an exciting opportunity to identify new circulating miRNAs, and validate those already known, as blood-based cancer markers. In addition, further functional analyses are required to characterize the cancer-specific role of these miRNAs.

## Supporting Information

Figure S1 **Example of quality control run of RNA isolated from plasma samples on an Agilent Bioanalyzer.**
(TIF)

Figure S2 **Box plot of global miRNA expression levels in the pairs of plasma samples collected before and after lung tumor surgery, before (upper panel) and after (lower panel) normalization.** Each box plot represents miRNA expression for one cDNA sample (typically two cDNA samples per patient). Values of C_t_ for the total set of 93 miRNAs were analyzed using BRB-ArrayTools software. Pairs of samples with abnormal values (marked with asterisks) were excluded from further analysis.
(TIF)

Table S1 **Summary for the patients participated in the study.**
(XLSX)

Table S2 **Results of additional validation for selected miRNAs.** Mean Ct values are shown for pre- and post-operative samples. “-” indicates no detection (Ct>42).
(XLSX)
