# Supplementary figures and images for: Comparisons of microRNA Patterns in Plasma before and after Tumor Removal Reveal New Biomarkers of Lung Squamous Cell Carcinoma
Source: PLoS One. 2013 Oct 9;8(10):e78649. doi: 10.1371/journal.pone.0078649 (PMC3793941; doi:10.1371/journal.pone.0078649)

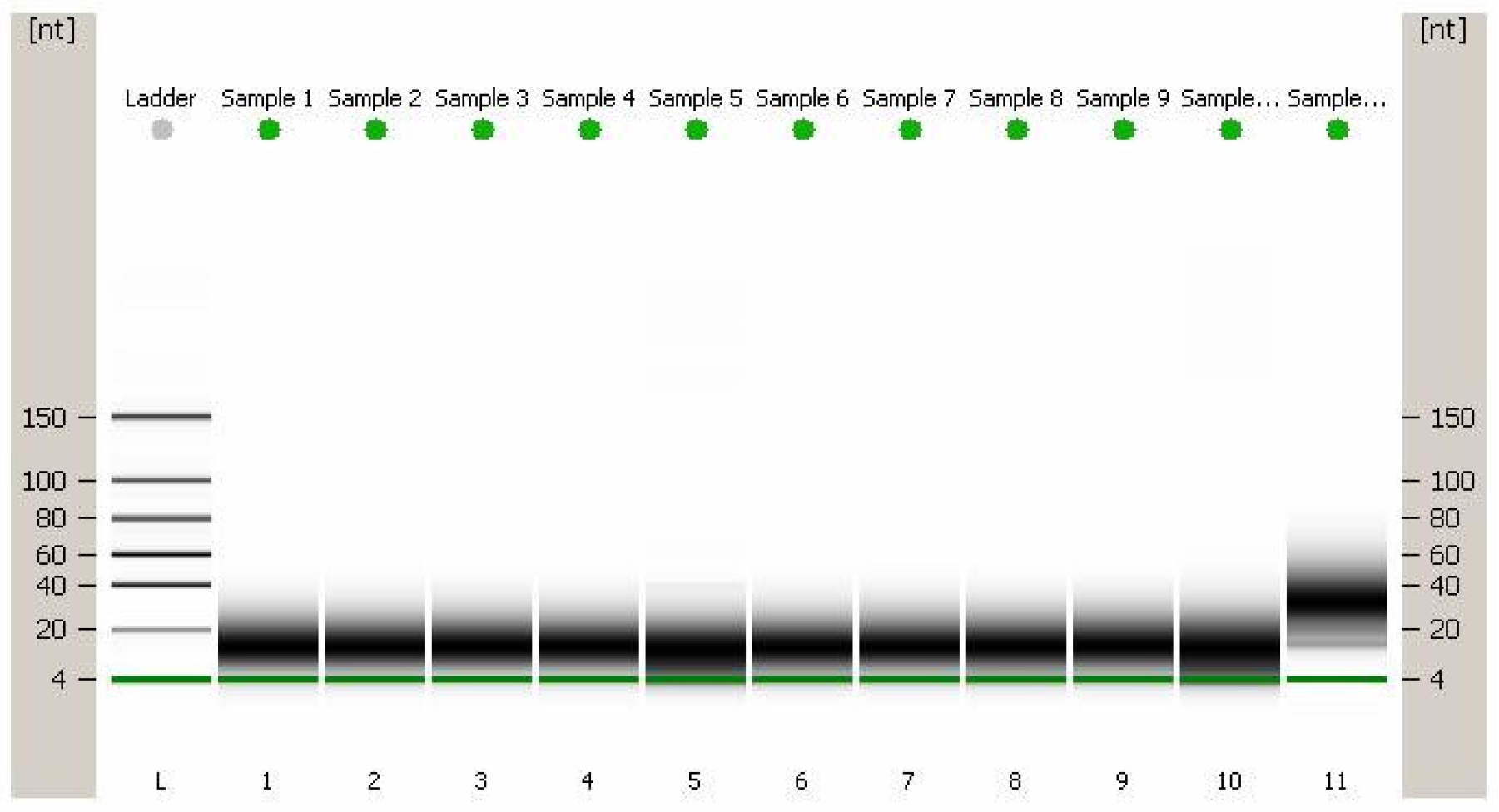

Supplement: Figure S1 — Example of quality control run of RNA isolated from plasma samples on an Agilent Bioanalyzer. (TIF) [file pone.0078649.s001.tif]

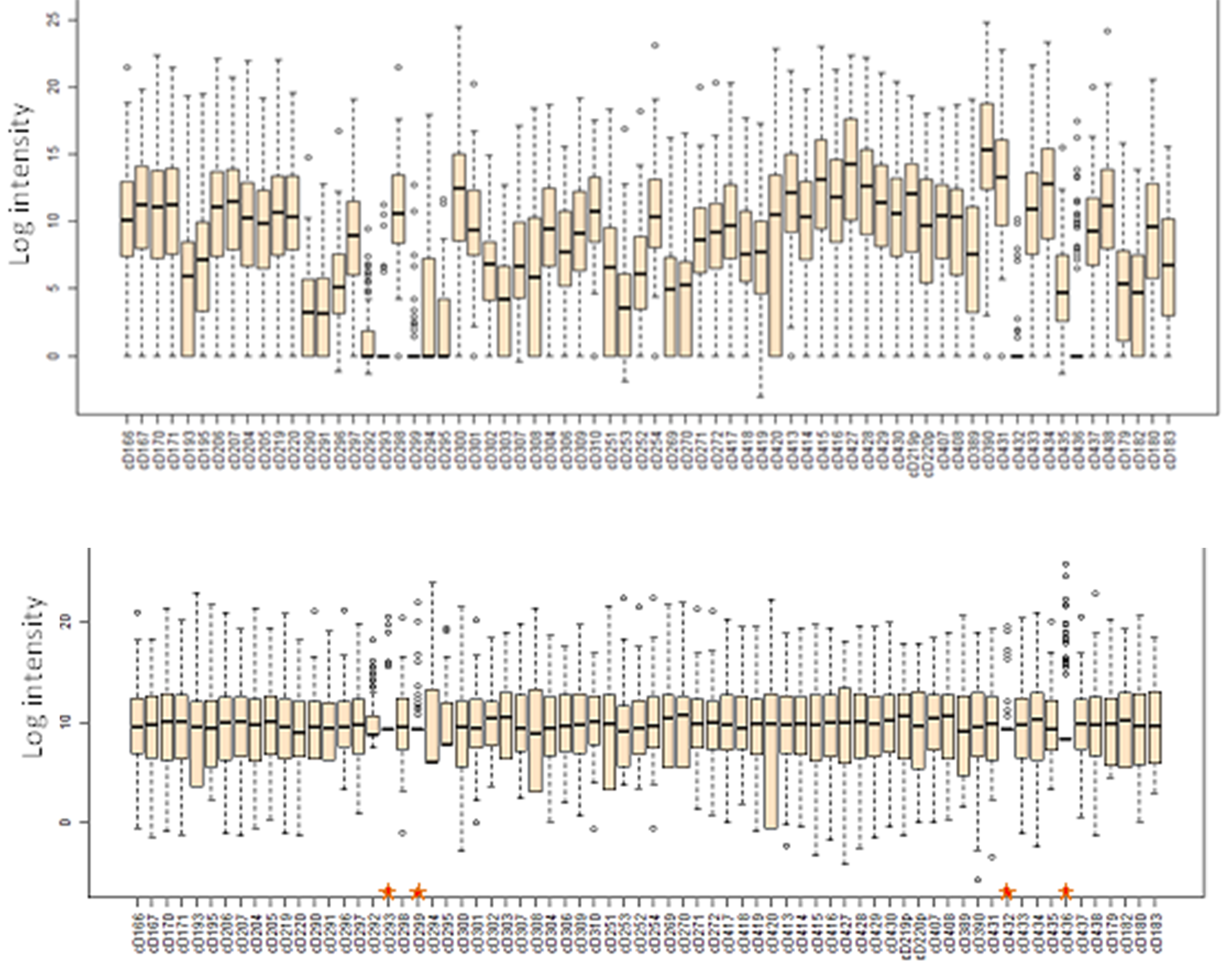

Supplement: Figure S2 — Box plot of global miRNA expression levels in the pairs of plasma samples collected before and after lung tumor surgery, before (upper panel) and after (lower panel) normalization. Each box plot represents miRNA expression for one cDNA sample (typically two cDNA samples per patient). Values of Ct for the total set of 93 miRNAs were analyzed using BRB-ArrayTools software. Pairs of samples with abnormal values (marked with asterisks) were excluded from further analysis. (TIF) [file pone.0078649.s002.tif]
